# Bis{[μ-bis­(diphenyl­phosphan­yl)methane-1:2κ^2^ *P*:*P*′]nona­carbonyl-1κ^3^ *C*,2κ^3^ *C*,3κ^3^ *C*-[(4-methyl­sulfanylphen­yl)diphenyl­phosphane-3κ*P*]-*triangulo*-triruthen­ium(0)} dichloro­methane monosolvate

**DOI:** 10.1107/S1600536811000778

**Published:** 2011-01-15

**Authors:** Omar bin Shawkataly, Imthyaz Ahmed Khan, H. A. Hafiz Malik, Chin Sing Yeap, Hoong-Kun Fun

**Affiliations:** a Chemical Sciences Programme, School of Distance Education, Universiti Sains Malaysia, 11800 USM, Penang, Malaysia; b X-ray Crystallography Unit, School of Physics, Universiti Sains Malaysia, 11800 USM, Penang, Malaysia

## Abstract

The asymmetric unit of the title *triangulo*-triruthenium compound, 2[Ru_3_(C_25_H_22_P_2_)(C_19_H_17_PS)(CO)_9_]·CH_2_Cl_2_, contains one *triangulo*-triruthenium complex mol­ecule and one half-mol­ecule of the dichloro­methane solvent. The dichloro­methane solvent mol­ecule lies across a crystallographic inversion center leading to the mol­ecule being disordered over two positions of equal occupancy. The bis­(diphenyl­phosphan­yl)methane ligand bridges an Ru—Ru bond and the monodentate phosphane ligand bonds to the third Ru atom. All phosphane ligands are equatorial with respect to the Ru_3_ triangle. In addition, each Ru atom carries one equatorial and two axial terminal carbonyl ligands. The three phosphane-substituted benzene rings make dihedral angles of 87.18 (11), 59.59 (10) and 89.28 (11)° with each other. The dihedral angles between the two benzene rings are 78.48 (11) and 87.58 (11)° for the two diphenyl­phosphanyl groups. In the crystal, the mol­ecules are stacked along the *a* axis. Weak inter­molecular C—H⋯π inter­actions stabilize the crystal structure.

## Related literature

For general background to *triangulo*-triruthenium derivatives, see: Bruce *et al.* (1985 ▶, 1988*a* ▶,*b* ▶). For related structures, see: Shawkataly *et al.* (1998 ▶, 2004 ▶, 2010*a* ▶,*b* ▶). For the synthesis of Ru_3_(CO)_10_(μ-Ph_2_PCH_2_PPh_2_), see: Bruce *et al.* (1983 ▶) and for that of 4-methyl­thio­phenyl­diphenylphosphine, see: Fuhr *et al.* (2002 ▶). For the stability of the temperature controller used in the data collection, see: Cosier & Glazer (1986 ▶).
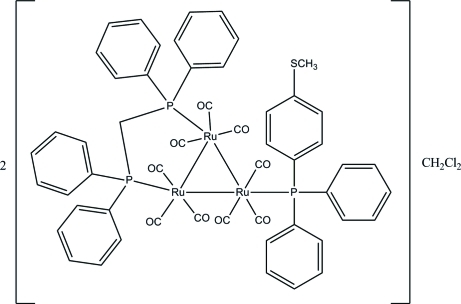


## Experimental

### 

#### Crystal data

2[Ru_3_(C_25_H_22_P_2_)(C_19_H_17_PS)(CO)_9_]·CH_2_Cl_2_
*M* *_r_* = 2580.97
Triclinic, 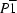

*a* = 10.7125 (1) Å
*b* = 12.4639 (1) Å
*c* = 20.0660 (2) Å
α = 96.260 (1)°
β = 104.180 (1)°
γ = 102.900 (1)°
*V* = 2493.72 (4) Å^3^
*Z* = 1
Mo *K*α radiation
μ = 1.15 mm^−1^
*T* = 100 K
0.22 × 0.18 × 0.11 mm

#### Data collection

Bruker SMART APEXII CCD area-detector diffractometer
Absorption correction: multi-scan (*SADABS*; Bruker, 2009 ▶) *T*_min_ = 0.784, *T*_max_ = 0.882
87820 measured reflections
21813 independent reflections
17718 reflections with *I* > 2σ(*I*)
*R*_int_ = 0.043

#### Refinement

*R*[*F* ^2^ > 2σ(*F* ^2^)] = 0.036
*wR*(*F* ^2^) = 0.085
*S* = 1.01
21813 reflections
640 parameters
H-atom parameters constrained
Δρ_max_ = 2.74 e Å^−3^
Δρ_min_ = −1.71 e Å^−3^

### 

Data collection: *APEX2* (Bruker, 2009 ▶); cell refinement: *SAINT* (Bruker, 2009 ▶); data reduction: *SAINT*; program(s) used to solve structure: *SHELXTL* (Sheldrick, 2008 ▶); program(s) used to refine structure: *SHELXTL*; molecular graphics: *SHELXTL*; software used to prepare material for publication: *SHELXTL* and *PLATON* (Spek, 2009 ▶).

## Supplementary Material

Crystal structure: contains datablocks global, I. DOI: 10.1107/S1600536811000778/sj5089sup1.cif

Structure factors: contains datablocks I. DOI: 10.1107/S1600536811000778/sj5089Isup2.hkl

Additional supplementary materials:  crystallographic information; 3D view; checkCIF report

## Figures and Tables

**Table 1:** Hydrogen-bond geometry (Å, °) *Cg*1, *Cg*2 and *Cg*3 are the centroids of the C26–C31, C14–C19 and C7–C12 benzene rings, respectively.

*D*—H⋯*A*	*D*—H	H⋯*A*	*D*⋯*A*	*D*—H⋯*A*
C9—H9*A*⋯*Cg*1^i^	0.93	2.83	3.550 (2)	135
C12—H12*A*⋯*Cg*2	0.93	2.98	3.743 (2)	140
C16—H16*A*⋯*Cg*1^ii^	0.93	2.90	3.696 (2)	145
C22—H22*A*⋯*Cg*3^iii^	0.93	2.99	3.708 (3)	136
C34—H34*A*⋯*Cg*2^iv^	0.93	2.98	3.850 (3)	156

Symmetry codes: (i) 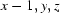; (ii) 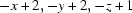; (iii) 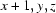; (iv) 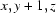.

**Fig. 1.**
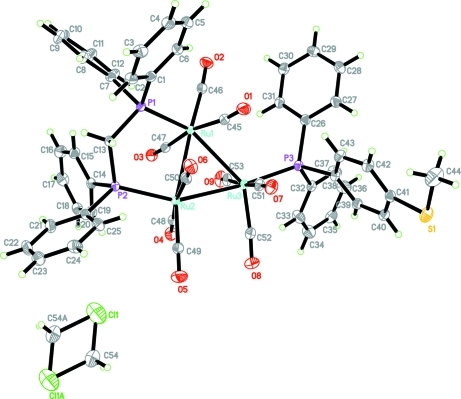
The molecular structure of the title compound with 50% probability ellipsoids for non-H atoms. Atoms with suffix A are generated by the symmetry operation (-x, 2 - y, -z).

**Fig. 2.**
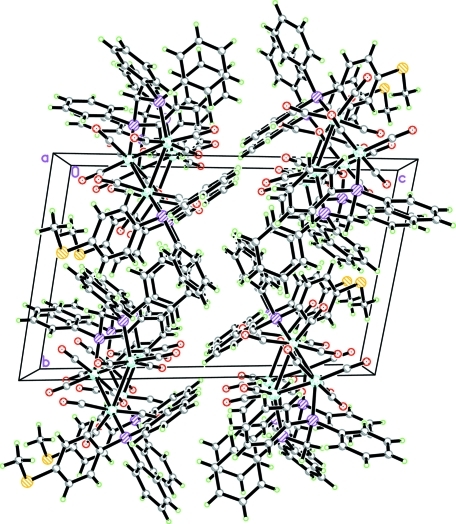
The crystal packing of the title compound, viewed down the a axis, showing the molecules stacked down a axis. Solvent molecule have been omitted for clarity.

## supplementary crystallographic information

### Comment

A large number of substituted derivatives of the type Ru_3_(CO)_12-n_*L*_n_ (*L* = group 15 ligand) have been reported (Bruce *et al.*, 1985, 1988*a*,*b*). As part of our study on the substitution of transition metal-carbonyl clusters with mixed-ligand complexes, we have published several structures of *triangulo*-triruthenium-carbonyl clusters containing mixed P/As and P/Sb ligands (Shawkataly *et al.*, 1998, 2004, 2010*a*,*b*). Herein we report the synthesis and structure of the title compound.

The asymmetric unit of title compound consists of one molecule of *triangulo*-triruthenium complex and one half-molecule of dichloromethane solvent (Fig. 1). The dichloromethane solvent lies across a crystallographic inversion center (symmetry code: -*x*, 2 - *y*, -*z*) leading to disorder of this solvent molecule over two positions. The title compound is very similar to those found in related structures (Shawkataly *et al.*, 2010*a*, *b*) with comparable cell parameters and similarly disordered dichloromethane solvent. The bis(diphenylphosphanyl)methane ligand bridges the Ru1–Ru2 bond and the monodentate phosphane ligand bonds to the Ru3 atom. All phosphane ligands are equatorial with respect to the Ru_3_ triangle. Additionally, each Ru atom carries one equatorial and two axial terminal carbonyl ligands. The three phosphane-substituted benzene rings make dihedral angles (C26–C31/C32–C37, C26–C31/C38–C43 and C32–C37/C38–C43) of 87.18 (11), 59.59 (10) and 89.28 (11)° with each other respectively. The dihedral angles between the two benzene rings (C1–C6/C7–C12 and C14–C19/C20–C25) are 78.48 (11) and 87.58 (11)° for the two diphenylphosphanyl groups respectively. The torsion angle of the methylthio group (C44–S1–C41–C42) is -14.1 (2)°.

In the crystal packing, the molecules are stacked along *a* axis (Fig. 2). Weak intermolecular C—H···π interactions (Table 1) stabilize the crystal structure.

### Experimental

All manipulations were performed under a dry oxygen-free nitrogen atmosphere using standard Schlenk techniques. All solvents were dried over sodium and distilled from sodium benzophenone ketyl under dry oxygen free nitrogen. 4-Methylthiophenyldiphenylphosphane (Fuhr *et al.*, 2002) and Ru_3_(CO)_10_(µ-Ph_2_PCH_2_PPh_2_) (Bruce *et al.*, 1983) was prepared by reported procedure. The title compound was obtained by refluxing equimolar quantities of Ru_3_(CO)_10_(µ-Ph_2_PCH_2_PPh_2_) and 4-methylthiophenyldiphenylphosphane in hexane under nitrogen atmosphere. Crystals suitable for X-ray diffraction were grown by slow solvent / solvent diffusion of CH_3_OH into CH_2_Cl_2_.

### Refinement

All hydrogen atoms were positioned geometrically and refined using a riding model with C—H = 0.93–0.97 Å and *U*_iso_(H) = 1.2 or 1.5 *U*_eq_(C). A rotating group model was applied for the methyl group. The maximum and minimum residual electron density peaks of 2.74 and -1.71 e Å^-3^ were located 0.66 Å and 0.36 Å from the Ru1 and Cl1 atoms, respectively.

### Crystal data

**Table:**

2[Ru_3_(C_25_H_22_P_2_)(C_19_H_17_PS)(CO)_9_]·CH_2_Cl_2_	*Z* = 1
*M**_r_* = 2580.97	*F*(000) = 1286
Triclinic, *P*1	*D*_x_ = 1.719 Mg m^−^^3^
Hall symbol: -P 1	Mo *K*α radiation, λ = 0.71073 Å
*a* = 10.7125 (1) Å	Cell parameters from 9836 reflections
*b* = 12.4639 (1) Å	θ = 2.3–38.1°
*c* = 20.0660 (2) Å	µ = 1.15 mm^−^^1^
α = 96.260 (1)°	*T* = 100 K
β = 104.180 (1)°	Block, brown
γ = 102.900 (1)°	0.22 × 0.18 × 0.11 mm
*V* = 2493.72 (4) Å^3^	

### Data collection

**Table:**

Bruker SMART APEXII CCD area-detector diffractometer	21813 independent reflections
Radiation source: fine-focus sealed tube	17718 reflections with *I* > 2σ(*I*)
graphite	*R*_int_ = 0.043
φ and ω scans	θ_max_ = 35.0°, θ_min_ = 1.7°
Absorption correction: multi-scan (*SADABS*; Bruker, 2009)	*h* = −17→17
*T*_min_ = 0.784, *T*_max_ = 0.882	*k* = −20→20
87820 measured reflections	*l* = −32→32

### Refinement

**Table:**

Refinement on *F*^2^	Primary atom site location: structure-invariant direct methods
Least-squares matrix: full	Secondary atom site location: difference Fourier map
*R*[*F*^2^ > 2σ(*F*^2^)] = 0.036	Hydrogen site location: inferred from neighbouring sites
*wR*(*F*^2^) = 0.085	H-atom parameters constrained
*S* = 1.01	*w* = 1/[σ^2^(*F*_o_^2^) + (0.033*P*)^2^ + 3.2031*P*] where *P* = (*F*_o_^2^ + 2*F*_c_^2^)/3
21813 reflections	(Δ/σ)_max_ = 0.004
640 parameters	Δρ_max_ = 2.74 e Å^−^^3^
0 restraints	Δρ_min_ = −1.71 e Å^−^^3^

### Special details

**Table:**

Experimental. The crystal was placed in the cold stream of an Oxford Cryosystems Cobra open-flow nitrogen cryostat (Cosier & Glazer, 1986) operating at 100.0 (1) K.
Geometry. All e.s.d.'s (except the e.s.d. in the dihedral angle between two l.s. planes) are estimated using the full covariance matrix. The cell e.s.d.'s are taken into account individually in the estimation of e.s.d.'s in distances, angles and torsion angles; correlations between e.s.d.'s in cell parameters are only used when they are defined by crystal symmetry. An approximate (isotropic) treatment of cell e.s.d.'s is used for estimating e.s.d.'s involving l.s. planes.
Refinement. Refinement of *F*^2^ against ALL reflections. The weighted *R*-factor *wR* and goodness of fit *S* are based on *F*^2^, conventional *R*-factors *R* are based on *F*, with *F* set to zero for negative *F*^2^. The threshold expression of *F*^2^ > σ(*F*^2^) is used only for calculating *R*-factors(gt) *etc*. and is not relevant to the choice of reflections for refinement. *R*-factors based on *F*^2^ are statistically about twice as large as those based on *F*, and *R*- factors based on ALL data will be even larger.

### Fractional atomic coordinates and isotropic or equivalent isotropic displacement parameters (Å^2^)

**Table:**

	*x*	*y*	*z*	*U*_iso_*/*U*_eq_	Occ. (<1)
Ru1	0.837948 (15)	1.157405 (12)	0.261089 (8)	0.01366 (3)	
Ru2	0.594161 (15)	0.998567 (12)	0.186830 (8)	0.01233 (3)	
Ru3	0.780393 (15)	0.930855 (12)	0.290459 (8)	0.01330 (3)	
P1	0.43487 (5)	0.83385 (4)	0.17996 (2)	0.01279 (8)	
P2	0.67393 (5)	0.74300 (4)	0.24661 (3)	0.01323 (8)	
P3	1.05584 (5)	1.26909 (4)	0.31731 (3)	0.01495 (9)	
S1	1.32009 (6)	1.43275 (5)	0.08033 (3)	0.02441 (11)	
O1	0.91609 (17)	1.10604 (14)	0.12550 (8)	0.0237 (3)	
O2	0.70316 (18)	1.32691 (14)	0.19956 (9)	0.0261 (3)	
O3	0.77257 (18)	1.20410 (15)	0.40045 (9)	0.0284 (4)	
O4	0.71518 (16)	0.87246 (14)	0.08966 (8)	0.0230 (3)	
O5	0.49337 (16)	1.14768 (13)	0.28241 (9)	0.0233 (3)	
O6	0.46309 (19)	1.09054 (16)	0.06142 (9)	0.0307 (4)	
O7	0.98385 (17)	0.92717 (15)	0.20611 (10)	0.0270 (3)	
O8	0.97139 (17)	0.88439 (15)	0.41640 (9)	0.0250 (3)	
O9	0.59365 (16)	0.97317 (13)	0.37958 (8)	0.0219 (3)	
C1	0.3005 (2)	0.78383 (16)	0.09879 (10)	0.0159 (3)	
C2	0.3332 (2)	0.78725 (18)	0.03562 (10)	0.0196 (4)	
H2A	0.4211	0.8169	0.0359	0.024*	
C3	0.2361 (2)	0.74697 (19)	−0.02733 (11)	0.0237 (4)	
H3A	0.2591	0.7485	−0.0691	0.028*	
C4	0.1040 (2)	0.70415 (19)	−0.02823 (12)	0.0258 (4)	
H4A	0.0385	0.6781	−0.0705	0.031*	
C5	0.0704 (2)	0.7004 (2)	0.03363 (13)	0.0274 (5)	
H5A	−0.0179	0.6714	0.0329	0.033*	
C6	0.1681 (2)	0.73989 (19)	0.09747 (11)	0.0222 (4)	
H6A	0.1448	0.7369	0.1391	0.027*	
C7	0.35137 (19)	0.82431 (16)	0.24873 (10)	0.0152 (3)	
C8	0.2855 (2)	0.90548 (17)	0.26281 (11)	0.0181 (3)	
H8A	0.2781	0.9593	0.2344	0.022*	
C9	0.2311 (2)	0.90620 (19)	0.31900 (11)	0.0214 (4)	
H9A	0.1885	0.9611	0.3284	0.026*	
C10	0.2401 (2)	0.8252 (2)	0.36132 (12)	0.0254 (4)	
H10A	0.2047	0.8266	0.3993	0.031*	
C11	0.3017 (2)	0.7428 (2)	0.34666 (12)	0.0242 (4)	
H11A	0.3066	0.6879	0.3744	0.029*	
C12	0.3567 (2)	0.74156 (17)	0.29038 (11)	0.0186 (4)	
H12A	0.3971	0.6853	0.2805	0.022*	
C13	0.51207 (19)	0.71602 (15)	0.18003 (10)	0.0145 (3)	
H13A	0.5248	0.6977	0.1343	0.017*	
H13B	0.4512	0.6514	0.1878	0.017*	
C14	0.63549 (19)	0.64811 (16)	0.30693 (10)	0.0146 (3)	
C15	0.6455 (2)	0.68838 (17)	0.37605 (10)	0.0186 (4)	
H15A	0.6775	0.7647	0.3932	0.022*	
C16	0.6080 (2)	0.61558 (18)	0.41975 (11)	0.0225 (4)	
H16A	0.6139	0.6435	0.4656	0.027*	
C17	0.5619 (2)	0.50136 (18)	0.39500 (12)	0.0235 (4)	
H17A	0.5359	0.4528	0.4240	0.028*	
C18	0.5550 (2)	0.45995 (18)	0.32651 (12)	0.0226 (4)	
H18A	0.5265	0.3833	0.3102	0.027*	
C19	0.5903 (2)	0.53249 (17)	0.28256 (11)	0.0184 (3)	
H19A	0.5841	0.5043	0.2367	0.022*	
C20	0.7655 (2)	0.66612 (16)	0.20189 (11)	0.0167 (3)	
C21	0.8994 (2)	0.67841 (17)	0.23616 (12)	0.0199 (4)	
H21A	0.9381	0.7256	0.2791	0.024*	
C22	0.9748 (2)	0.62100 (19)	0.20680 (13)	0.0235 (4)	
H22A	1.0641	0.6303	0.2298	0.028*	
C23	0.9174 (2)	0.54943 (19)	0.14297 (13)	0.0240 (4)	
H23A	0.9682	0.5108	0.1233	0.029*	
C24	0.7849 (2)	0.53592 (19)	0.10887 (12)	0.0227 (4)	
H24A	0.7465	0.4880	0.0662	0.027*	
C25	0.7084 (2)	0.59360 (17)	0.13801 (11)	0.0192 (4)	
H25A	0.6190	0.5838	0.1149	0.023*	
C26	1.1750 (2)	1.21173 (16)	0.37579 (10)	0.0168 (3)	
C27	1.3092 (2)	1.22971 (18)	0.37581 (11)	0.0202 (4)	
H27A	1.3403	1.2721	0.3451	0.024*	
C28	1.3962 (2)	1.18506 (19)	0.42107 (11)	0.0220 (4)	
H28A	1.4846	1.1975	0.4203	0.026*	
C29	1.3514 (2)	1.12195 (19)	0.46744 (11)	0.0216 (4)	
H29A	1.4098	1.0923	0.4978	0.026*	
C30	1.2194 (2)	1.10329 (18)	0.46837 (11)	0.0203 (4)	
H30A	1.1892	1.0606	0.4992	0.024*	
C31	1.1317 (2)	1.14823 (17)	0.42321 (11)	0.0190 (4)	
H31A	1.0435	1.1359	0.4246	0.023*	
C32	1.0630 (2)	1.40060 (16)	0.37212 (10)	0.0173 (3)	
C33	0.9553 (2)	1.4472 (2)	0.36079 (13)	0.0270 (5)	
H33A	0.8775	1.4100	0.3263	0.032*	
C34	0.9608 (3)	1.5484 (2)	0.39975 (13)	0.0279 (5)	
H34A	0.8878	1.5788	0.3905	0.033*	
C35	1.0748 (2)	1.60378 (18)	0.45236 (11)	0.0234 (4)	
H35A	1.0785	1.6708	0.4791	0.028*	
C36	1.1832 (3)	1.5583 (2)	0.46469 (13)	0.0304 (5)	
H36A	1.2602	1.5953	0.4998	0.036*	
C37	1.1781 (2)	1.45736 (19)	0.42489 (12)	0.0257 (4)	
H37A	1.2517	1.4278	0.4336	0.031*	
C38	1.1454 (2)	1.31731 (16)	0.25485 (10)	0.0166 (3)	
C39	1.1601 (2)	1.42591 (17)	0.24096 (11)	0.0179 (3)	
H39A	1.1326	1.4773	0.2674	0.022*	
C40	1.2153 (2)	1.45860 (17)	0.18801 (11)	0.0190 (4)	
H40A	1.2223	1.5308	0.1787	0.023*	
C41	1.2601 (2)	1.38383 (17)	0.14886 (10)	0.0175 (3)	
C42	1.2494 (2)	1.27573 (17)	0.16348 (11)	0.0197 (4)	
H42A	1.2810	1.2256	0.1385	0.024*	
C43	1.1912 (2)	1.24295 (17)	0.21545 (11)	0.0199 (4)	
H43A	1.1827	1.1703	0.2241	0.024*	
C44	1.3978 (3)	1.3274 (2)	0.05386 (16)	0.0389 (7)	
H44A	1.4340	1.3473	0.0163	0.058*	
H44B	1.3327	1.2566	0.0386	0.058*	
H44C	1.4682	1.3224	0.0926	0.058*	
C45	0.8849 (2)	1.11987 (16)	0.17560 (11)	0.0180 (3)	
C46	0.7595 (2)	1.26603 (17)	0.22321 (11)	0.0185 (4)	
C47	0.7932 (2)	1.17875 (18)	0.34831 (11)	0.0206 (4)	
C48	0.6764 (2)	0.92090 (17)	0.12839 (10)	0.0168 (3)	
C49	0.5344 (2)	1.09059 (16)	0.25009 (10)	0.0168 (3)	
C50	0.5111 (2)	1.05834 (17)	0.10979 (11)	0.0186 (4)	
C51	0.9068 (2)	0.93514 (17)	0.23563 (11)	0.0195 (4)	
C52	0.9029 (2)	0.90592 (17)	0.36893 (11)	0.0181 (3)	
C53	0.6593 (2)	0.95889 (16)	0.34399 (10)	0.0174 (3)	
Cl1	0.13190 (9)	1.00231 (8)	0.04461 (5)	0.0540 (2)	
C54	0.0346 (6)	1.0646 (4)	−0.0049 (3)	0.0318 (10)	0.50
H54A	0.0745	1.0841	−0.0413	0.038*	0.50
H54B	0.0376	1.1334	0.0229	0.038*	0.50

### Atomic displacement parameters (Å^2^)

**Table:**

	*U*^11^	*U*^22^	*U*^33^	*U*^12^	*U*^13^	*U*^23^
Ru1	0.01402 (7)	0.01148 (6)	0.01443 (6)	0.00121 (5)	0.00397 (5)	0.00218 (5)
Ru2	0.01216 (6)	0.01174 (6)	0.01320 (6)	0.00280 (5)	0.00398 (5)	0.00220 (4)
Ru3	0.01274 (6)	0.01153 (6)	0.01471 (6)	0.00227 (5)	0.00285 (5)	0.00247 (5)
P1	0.0122 (2)	0.01268 (19)	0.01312 (19)	0.00267 (16)	0.00349 (16)	0.00181 (15)
P2	0.0137 (2)	0.01182 (19)	0.0146 (2)	0.00328 (16)	0.00476 (16)	0.00251 (15)
P3	0.0148 (2)	0.0129 (2)	0.0163 (2)	0.00181 (16)	0.00454 (17)	0.00195 (16)
S1	0.0311 (3)	0.0224 (2)	0.0211 (2)	0.0038 (2)	0.0121 (2)	0.00478 (19)
O1	0.0277 (8)	0.0232 (7)	0.0209 (7)	0.0041 (6)	0.0103 (6)	0.0039 (6)
O2	0.0330 (9)	0.0218 (7)	0.0262 (8)	0.0120 (7)	0.0074 (7)	0.0068 (6)
O3	0.0319 (9)	0.0285 (8)	0.0208 (7)	−0.0019 (7)	0.0118 (7)	−0.0032 (6)
O4	0.0207 (7)	0.0258 (8)	0.0223 (7)	0.0060 (6)	0.0080 (6)	−0.0011 (6)
O5	0.0229 (8)	0.0203 (7)	0.0278 (8)	0.0061 (6)	0.0107 (6)	−0.0011 (6)
O6	0.0338 (10)	0.0336 (9)	0.0269 (8)	0.0132 (8)	0.0049 (7)	0.0139 (7)
O7	0.0237 (8)	0.0314 (9)	0.0353 (9)	0.0136 (7)	0.0154 (7)	0.0159 (7)
O8	0.0211 (8)	0.0320 (9)	0.0230 (7)	0.0097 (6)	0.0031 (6)	0.0099 (6)
O9	0.0225 (8)	0.0237 (7)	0.0212 (7)	0.0060 (6)	0.0085 (6)	0.0049 (6)
C1	0.0153 (8)	0.0147 (8)	0.0160 (8)	0.0033 (6)	0.0020 (6)	0.0014 (6)
C2	0.0174 (9)	0.0244 (9)	0.0155 (8)	0.0050 (7)	0.0030 (7)	0.0011 (7)
C3	0.0276 (11)	0.0250 (10)	0.0156 (8)	0.0087 (8)	0.0007 (8)	0.0005 (7)
C4	0.0258 (11)	0.0223 (10)	0.0205 (9)	0.0015 (8)	−0.0040 (8)	0.0000 (8)
C5	0.0169 (10)	0.0306 (11)	0.0265 (11)	−0.0029 (8)	−0.0016 (8)	0.0070 (9)
C6	0.0176 (9)	0.0251 (10)	0.0203 (9)	−0.0005 (7)	0.0035 (7)	0.0062 (7)
C7	0.0138 (8)	0.0159 (8)	0.0157 (8)	0.0019 (6)	0.0061 (6)	0.0014 (6)
C8	0.0151 (8)	0.0185 (8)	0.0210 (9)	0.0045 (7)	0.0061 (7)	0.0019 (7)
C9	0.0178 (9)	0.0235 (10)	0.0230 (9)	0.0058 (7)	0.0078 (8)	−0.0009 (7)
C10	0.0228 (10)	0.0343 (12)	0.0218 (10)	0.0071 (9)	0.0116 (8)	0.0041 (8)
C11	0.0257 (11)	0.0292 (11)	0.0223 (10)	0.0076 (9)	0.0121 (8)	0.0106 (8)
C12	0.0197 (9)	0.0170 (8)	0.0215 (9)	0.0051 (7)	0.0090 (7)	0.0050 (7)
C13	0.0141 (8)	0.0132 (7)	0.0149 (7)	0.0033 (6)	0.0029 (6)	0.0007 (6)
C14	0.0145 (8)	0.0139 (7)	0.0172 (8)	0.0054 (6)	0.0053 (6)	0.0045 (6)
C15	0.0246 (10)	0.0146 (8)	0.0164 (8)	0.0047 (7)	0.0052 (7)	0.0029 (6)
C16	0.0308 (11)	0.0201 (9)	0.0162 (8)	0.0052 (8)	0.0067 (8)	0.0041 (7)
C17	0.0282 (11)	0.0199 (9)	0.0230 (10)	0.0031 (8)	0.0089 (8)	0.0088 (8)
C18	0.0274 (11)	0.0143 (8)	0.0253 (10)	0.0009 (7)	0.0097 (8)	0.0044 (7)
C19	0.0197 (9)	0.0160 (8)	0.0193 (8)	0.0033 (7)	0.0062 (7)	0.0027 (7)
C20	0.0183 (9)	0.0141 (8)	0.0209 (9)	0.0055 (7)	0.0094 (7)	0.0044 (6)
C21	0.0171 (9)	0.0175 (8)	0.0259 (10)	0.0047 (7)	0.0072 (8)	0.0040 (7)
C22	0.0179 (9)	0.0203 (9)	0.0353 (12)	0.0075 (8)	0.0100 (9)	0.0063 (8)
C23	0.0249 (10)	0.0208 (9)	0.0339 (11)	0.0108 (8)	0.0166 (9)	0.0063 (8)
C24	0.0275 (11)	0.0220 (9)	0.0220 (9)	0.0106 (8)	0.0100 (8)	0.0025 (7)
C25	0.0197 (9)	0.0195 (9)	0.0196 (9)	0.0080 (7)	0.0051 (7)	0.0029 (7)
C26	0.0161 (8)	0.0156 (8)	0.0175 (8)	0.0029 (6)	0.0041 (7)	0.0016 (6)
C27	0.0186 (9)	0.0218 (9)	0.0207 (9)	0.0046 (7)	0.0066 (7)	0.0049 (7)
C28	0.0185 (9)	0.0263 (10)	0.0224 (9)	0.0064 (8)	0.0081 (8)	0.0028 (8)
C29	0.0223 (10)	0.0253 (10)	0.0171 (8)	0.0091 (8)	0.0033 (7)	0.0030 (7)
C30	0.0234 (10)	0.0217 (9)	0.0154 (8)	0.0036 (8)	0.0063 (7)	0.0047 (7)
C31	0.0185 (9)	0.0178 (8)	0.0208 (9)	0.0029 (7)	0.0072 (7)	0.0038 (7)
C32	0.0192 (9)	0.0140 (8)	0.0181 (8)	0.0016 (7)	0.0063 (7)	0.0025 (6)
C33	0.0205 (10)	0.0242 (10)	0.0306 (11)	0.0054 (8)	0.0020 (9)	−0.0067 (8)
C34	0.0273 (11)	0.0229 (10)	0.0326 (12)	0.0091 (9)	0.0085 (9)	−0.0041 (9)
C35	0.0337 (12)	0.0157 (9)	0.0192 (9)	0.0032 (8)	0.0089 (8)	−0.0005 (7)
C36	0.0333 (13)	0.0224 (10)	0.0260 (11)	0.0054 (9)	−0.0033 (9)	−0.0056 (8)
C37	0.0239 (11)	0.0212 (10)	0.0262 (10)	0.0075 (8)	−0.0017 (8)	−0.0034 (8)
C38	0.0157 (8)	0.0149 (8)	0.0174 (8)	0.0010 (6)	0.0046 (7)	0.0020 (6)
C39	0.0177 (9)	0.0157 (8)	0.0211 (9)	0.0036 (7)	0.0068 (7)	0.0038 (7)
C40	0.0193 (9)	0.0158 (8)	0.0224 (9)	0.0037 (7)	0.0069 (7)	0.0047 (7)
C41	0.0159 (8)	0.0178 (8)	0.0164 (8)	0.0012 (7)	0.0033 (7)	0.0024 (6)
C42	0.0222 (10)	0.0170 (8)	0.0197 (9)	0.0038 (7)	0.0079 (7)	0.0004 (7)
C43	0.0225 (10)	0.0153 (8)	0.0215 (9)	0.0029 (7)	0.0071 (8)	0.0032 (7)
C44	0.0583 (19)	0.0320 (13)	0.0376 (14)	0.0156 (13)	0.0303 (14)	0.0062 (11)
C45	0.0181 (9)	0.0137 (8)	0.0204 (9)	0.0018 (7)	0.0043 (7)	0.0029 (6)
C46	0.0194 (9)	0.0172 (8)	0.0177 (8)	0.0020 (7)	0.0058 (7)	0.0022 (7)
C47	0.0178 (9)	0.0184 (9)	0.0224 (9)	−0.0010 (7)	0.0052 (7)	0.0023 (7)
C48	0.0140 (8)	0.0175 (8)	0.0170 (8)	0.0022 (6)	0.0029 (6)	0.0026 (6)
C49	0.0155 (8)	0.0160 (8)	0.0183 (8)	0.0031 (6)	0.0043 (7)	0.0036 (6)
C50	0.0176 (9)	0.0174 (8)	0.0209 (9)	0.0042 (7)	0.0058 (7)	0.0034 (7)
C51	0.0170 (9)	0.0185 (9)	0.0231 (9)	0.0038 (7)	0.0044 (7)	0.0087 (7)
C52	0.0164 (9)	0.0162 (8)	0.0216 (9)	0.0035 (7)	0.0059 (7)	0.0029 (7)
C53	0.0183 (9)	0.0134 (8)	0.0185 (8)	0.0027 (7)	0.0026 (7)	0.0033 (6)
Cl1	0.0426 (4)	0.0467 (4)	0.0630 (5)	0.0157 (3)	0.0052 (4)	−0.0177 (4)
C54	0.038 (3)	0.025 (2)	0.036 (3)	0.007 (2)	0.019 (2)	0.0016 (19)

### Geometric parameters (Å, °)

**Table:**

Ru1—C46	1.880 (2)	C15—C16	1.392 (3)
Ru1—C47	1.931 (2)	C15—H15A	0.9300
Ru1—C45	1.941 (2)	C16—C17	1.389 (3)
Ru1—P3	2.3612 (5)	C16—H16A	0.9300
Ru1—Ru2	2.8463 (2)	C17—C18	1.392 (3)
Ru1—Ru3	2.9093 (2)	C17—H17A	0.9300
Ru2—C50	1.900 (2)	C18—C19	1.387 (3)
Ru2—C48	1.931 (2)	C18—H18A	0.9300
Ru2—C49	1.935 (2)	C19—H19A	0.9300
Ru2—P1	2.3248 (5)	C20—C21	1.396 (3)
Ru2—Ru3	2.8493 (2)	C20—C25	1.396 (3)
Ru3—C52	1.891 (2)	C21—C22	1.382 (3)
Ru3—C51	1.939 (2)	C21—H21A	0.9300
Ru3—C53	1.940 (2)	C22—C23	1.391 (3)
Ru3—P2	2.3288 (5)	C22—H22A	0.9300
P1—C7	1.8198 (19)	C23—C24	1.380 (3)
P1—C1	1.828 (2)	C23—H23A	0.9300
P1—C13	1.8392 (19)	C24—C25	1.392 (3)
P2—C20	1.830 (2)	C24—H24A	0.9300
P2—C14	1.8364 (19)	C25—H25A	0.9300
P2—C13	1.847 (2)	C26—C31	1.401 (3)
P3—C38	1.830 (2)	C26—C27	1.404 (3)
P3—C26	1.837 (2)	C27—C28	1.389 (3)
P3—C32	1.847 (2)	C27—H27A	0.9300
S1—C41	1.765 (2)	C28—C29	1.387 (3)
S1—C44	1.803 (3)	C28—H28A	0.9300
O1—C45	1.142 (3)	C29—C30	1.386 (3)
O2—C46	1.144 (3)	C29—H29A	0.9300
O3—C47	1.147 (3)	C30—C31	1.395 (3)
O4—C48	1.144 (2)	C30—H30A	0.9300
O5—C49	1.140 (2)	C31—H31A	0.9300
O6—C50	1.140 (3)	C32—C33	1.386 (3)
O7—C51	1.142 (3)	C32—C37	1.396 (3)
O8—C52	1.148 (3)	C33—C34	1.390 (3)
O9—C53	1.145 (3)	C33—H33A	0.9300
C1—C6	1.395 (3)	C34—C35	1.384 (3)
C1—C2	1.398 (3)	C34—H34A	0.9300
C2—C3	1.384 (3)	C35—C36	1.384 (4)
C2—H2A	0.9300	C35—H35A	0.9300
C3—C4	1.391 (3)	C36—C37	1.397 (3)
C3—H3A	0.9300	C36—H36A	0.9300
C4—C5	1.377 (3)	C37—H37A	0.9300
C4—H4A	0.9300	C38—C39	1.394 (3)
C5—C6	1.398 (3)	C38—C43	1.400 (3)
C5—H5A	0.9300	C39—C40	1.393 (3)
C6—H6A	0.9300	C39—H39A	0.9300
C7—C8	1.398 (3)	C40—C41	1.393 (3)
C7—C12	1.399 (3)	C40—H40A	0.9300
C8—C9	1.390 (3)	C41—C42	1.396 (3)
C8—H8A	0.9300	C42—C43	1.393 (3)
C9—C10	1.393 (3)	C42—H42A	0.9300
C9—H9A	0.9300	C43—H43A	0.9300
C10—C11	1.381 (3)	C44—H44A	0.9600
C10—H10A	0.9300	C44—H44B	0.9600
C11—C12	1.396 (3)	C44—H44C	0.9600
C11—H11A	0.9300	Cl1—C54	1.643 (6)
C12—H12A	0.9300	Cl1—C54^i^	1.734 (6)
C13—H13A	0.9700	C54—C54^i^	1.671 (10)
C13—H13B	0.9700	C54—Cl1^i^	1.734 (6)
C14—C15	1.392 (3)	C54—H54A	0.9600
C14—C19	1.403 (3)	C54—H54B	0.9600
			
C46—Ru1—C47	95.35 (9)	C14—C15—H15A	119.7
C46—Ru1—C45	90.57 (9)	C17—C16—C15	120.16 (19)
C47—Ru1—C45	173.67 (9)	C17—C16—H16A	119.9
C46—Ru1—P3	99.89 (6)	C15—C16—H16A	119.9
C47—Ru1—P3	89.12 (6)	C16—C17—C18	119.66 (19)
C45—Ru1—P3	92.08 (6)	C16—C17—H17A	120.2
C46—Ru1—Ru2	86.49 (6)	C18—C17—H17A	120.2
C47—Ru1—Ru2	96.00 (6)	C19—C18—C17	120.26 (19)
C45—Ru1—Ru2	82.10 (6)	C19—C18—H18A	119.9
P3—Ru1—Ru2	171.443 (15)	C17—C18—H18A	119.9
C46—Ru1—Ru3	143.73 (6)	C18—C19—C14	120.40 (19)
C47—Ru1—Ru3	78.03 (6)	C18—C19—H19A	119.8
C45—Ru1—Ru3	95.87 (6)	C14—C19—H19A	119.8
P3—Ru1—Ru3	115.437 (14)	C21—C20—C25	118.95 (18)
Ru2—Ru1—Ru3	59.332 (5)	C21—C20—P2	116.83 (15)
C50—Ru2—C48	90.33 (9)	C25—C20—P2	124.17 (16)
C50—Ru2—C49	91.23 (9)	C22—C21—C20	120.5 (2)
C48—Ru2—C49	172.45 (8)	C22—C21—H21A	119.7
C50—Ru2—P1	102.20 (6)	C20—C21—H21A	119.7
C48—Ru2—P1	90.72 (6)	C21—C22—C23	120.2 (2)
C49—Ru2—P1	96.16 (6)	C21—C22—H22A	119.9
C50—Ru2—Ru1	107.96 (6)	C23—C22—H22A	119.9
C48—Ru2—Ru1	93.64 (6)	C24—C23—C22	119.9 (2)
C49—Ru2—Ru1	78.85 (6)	C24—C23—H23A	120.1
P1—Ru2—Ru1	149.487 (14)	C22—C23—H23A	120.1
C50—Ru2—Ru3	164.65 (6)	C23—C24—C25	120.3 (2)
C48—Ru2—Ru3	79.85 (6)	C23—C24—H24A	119.8
C49—Ru2—Ru3	97.03 (6)	C25—C24—H24A	119.8
P1—Ru2—Ru3	89.791 (13)	C24—C25—C20	120.2 (2)
Ru1—Ru2—Ru3	61.435 (5)	C24—C25—H25A	119.9
C52—Ru3—C51	91.69 (9)	C20—C25—H25A	119.9
C52—Ru3—C53	92.83 (9)	C31—C26—C27	118.04 (19)
C51—Ru3—C53	168.20 (8)	C31—C26—P3	119.12 (16)
C52—Ru3—P2	95.90 (6)	C27—C26—P3	122.83 (15)
C51—Ru3—P2	93.45 (6)	C28—C27—C26	121.00 (19)
C53—Ru3—P2	96.92 (6)	C28—C27—H27A	119.5
C52—Ru3—Ru2	170.80 (6)	C26—C27—H27A	119.5
C51—Ru3—Ru2	93.48 (6)	C29—C28—C27	120.2 (2)
C53—Ru3—Ru2	80.69 (6)	C29—C28—H28A	119.9
P2—Ru3—Ru2	91.383 (14)	C27—C28—H28A	119.9
C52—Ru3—Ru1	115.11 (6)	C30—C29—C28	119.8 (2)
C51—Ru3—Ru1	74.82 (6)	C30—C29—H29A	120.1
C53—Ru3—Ru1	93.40 (6)	C28—C29—H29A	120.1
P2—Ru3—Ru1	146.748 (14)	C29—C30—C31	120.33 (19)
Ru2—Ru3—Ru1	59.233 (5)	C29—C30—H30A	119.8
C7—P1—C1	104.73 (9)	C31—C30—H30A	119.8
C7—P1—C13	105.47 (9)	C30—C31—C26	120.67 (19)
C1—P1—C13	99.66 (9)	C30—C31—H31A	119.7
C7—P1—Ru2	117.89 (6)	C26—C31—H31A	119.7
C1—P1—Ru2	117.46 (7)	C33—C32—C37	118.1 (2)
C13—P1—Ru2	109.54 (6)	C33—C32—P3	120.83 (16)
C20—P2—C14	99.53 (9)	C37—C32—P3	121.07 (17)
C20—P2—C13	102.11 (9)	C32—C33—C34	121.6 (2)
C14—P2—C13	102.64 (9)	C32—C33—H33A	119.2
C20—P2—Ru3	115.33 (7)	C34—C33—H33A	119.2
C14—P2—Ru3	119.45 (6)	C35—C34—C33	120.0 (2)
C13—P2—Ru3	115.15 (6)	C35—C34—H34A	120.0
C38—P3—C26	102.93 (9)	C33—C34—H34A	120.0
C38—P3—C32	103.06 (9)	C36—C35—C34	119.2 (2)
C26—P3—C32	102.38 (9)	C36—C35—H35A	120.4
C38—P3—Ru1	112.00 (7)	C34—C35—H35A	120.4
C26—P3—Ru1	120.02 (7)	C35—C36—C37	120.7 (2)
C32—P3—Ru1	114.46 (7)	C35—C36—H36A	119.7
C41—S1—C44	103.48 (12)	C37—C36—H36A	119.7
C6—C1—C2	118.96 (18)	C32—C37—C36	120.4 (2)
C6—C1—P1	122.73 (15)	C32—C37—H37A	119.8
C2—C1—P1	118.28 (15)	C36—C37—H37A	119.8
C3—C2—C1	120.7 (2)	C39—C38—C43	118.14 (18)
C3—C2—H2A	119.7	C39—C38—P3	121.15 (15)
C1—C2—H2A	119.7	C43—C38—P3	120.49 (15)
C2—C3—C4	120.0 (2)	C40—C39—C38	120.94 (19)
C2—C3—H3A	120.0	C40—C39—H39A	119.5
C4—C3—H3A	120.0	C38—C39—H39A	119.5
C5—C4—C3	119.9 (2)	C39—C40—C41	120.48 (19)
C5—C4—H4A	120.0	C39—C40—H40A	119.8
C3—C4—H4A	120.0	C41—C40—H40A	119.8
C4—C5—C6	120.4 (2)	C40—C41—C42	119.17 (18)
C4—C5—H5A	119.8	C40—C41—S1	116.22 (15)
C6—C5—H5A	119.8	C42—C41—S1	124.54 (16)
C1—C6—C5	120.0 (2)	C43—C42—C41	119.97 (19)
C1—C6—H6A	120.0	C43—C42—H42A	120.0
C5—C6—H6A	120.0	C41—C42—H42A	120.0
C8—C7—C12	118.82 (18)	C42—C43—C38	121.26 (19)
C8—C7—P1	119.05 (15)	C42—C43—H43A	119.4
C12—C7—P1	122.05 (15)	C38—C43—H43A	119.4
C9—C8—C7	120.3 (2)	S1—C44—H44A	109.5
C9—C8—H8A	119.8	S1—C44—H44B	109.5
C7—C8—H8A	119.8	H44A—C44—H44B	109.5
C8—C9—C10	120.4 (2)	S1—C44—H44C	109.5
C8—C9—H9A	119.8	H44A—C44—H44C	109.5
C10—C9—H9A	119.8	H44B—C44—H44C	109.5
C11—C10—C9	119.7 (2)	O1—C45—Ru1	174.94 (18)
C11—C10—H10A	120.2	O2—C46—Ru1	175.11 (19)
C9—C10—H10A	120.2	O3—C47—Ru1	172.24 (19)
C10—C11—C12	120.3 (2)	O4—C48—Ru2	174.34 (18)
C10—C11—H11A	119.9	O5—C49—Ru2	174.10 (19)
C12—C11—H11A	119.9	O6—C50—Ru2	176.5 (2)
C11—C12—C7	120.43 (19)	O7—C51—Ru3	172.32 (18)
C11—C12—H12A	119.8	O8—C52—Ru3	175.55 (18)
C7—C12—H12A	119.8	O9—C53—Ru3	174.99 (18)
P1—C13—P2	114.17 (10)	C54—Cl1—C54^i^	59.2 (3)
P1—C13—H13A	108.7	Cl1—C54—C54^i^	63.1 (4)
P2—C13—H13A	108.7	Cl1—C54—Cl1^i^	120.8 (3)
P1—C13—H13B	108.7	C54^i^—C54—Cl1^i^	57.7 (3)
P2—C13—H13B	108.7	Cl1—C54—H54A	106.9
H13A—C13—H13B	107.6	C54^i^—C54—H54A	126.1
C15—C14—C19	118.86 (17)	Cl1^i^—C54—H54A	107.0
C15—C14—P2	121.51 (15)	Cl1—C54—H54B	107.2
C19—C14—P2	119.60 (15)	C54^i^—C54—H54B	126.8
C16—C15—C14	120.63 (19)	Cl1^i^—C54—H54B	107.3
C16—C15—H15A	119.7	H54A—C54—H54B	107.0
			
C46—Ru1—Ru2—C50	24.83 (9)	C7—P1—C1—C2	176.39 (16)
C47—Ru1—Ru2—C50	119.85 (9)	C13—P1—C1—C2	−74.67 (18)
C45—Ru1—Ru2—C50	−66.23 (9)	Ru2—P1—C1—C2	43.42 (18)
Ru3—Ru1—Ru2—C50	−167.90 (7)	C6—C1—C2—C3	−0.5 (3)
C46—Ru1—Ru2—C48	116.36 (9)	P1—C1—C2—C3	177.81 (17)
C47—Ru1—Ru2—C48	−148.62 (9)	C1—C2—C3—C4	1.0 (3)
C45—Ru1—Ru2—C48	25.30 (9)	C2—C3—C4—C5	−1.0 (4)
C46—Ru1—Ru2—C49	−62.83 (9)	C3—C4—C5—C6	0.3 (4)
C47—Ru1—Ru2—C49	32.19 (9)	C2—C1—C6—C5	−0.2 (3)
C45—Ru1—Ru2—C49	−153.89 (9)	P1—C1—C6—C5	−178.36 (18)
Ru3—Ru1—Ru2—C49	104.44 (6)	C4—C5—C6—C1	0.2 (4)
C46—Ru1—Ru2—P1	−146.01 (7)	C1—P1—C7—C8	−77.05 (17)
C47—Ru1—Ru2—P1	−50.99 (7)	C13—P1—C7—C8	178.31 (16)
C45—Ru1—Ru2—P1	122.93 (7)	Ru2—P1—C7—C8	55.69 (18)
Ru3—Ru1—Ru2—P1	21.26 (3)	C1—P1—C7—C12	106.28 (18)
C46—Ru1—Ru2—Ru3	−167.27 (6)	C13—P1—C7—C12	1.63 (19)
C47—Ru1—Ru2—Ru3	−72.25 (7)	Ru2—P1—C7—C12	−120.99 (16)
C45—Ru1—Ru2—Ru3	101.67 (6)	C12—C7—C8—C9	2.6 (3)
C50—Ru2—Ru3—C51	−21.1 (2)	P1—C7—C8—C9	−174.22 (16)
C48—Ru2—Ru3—C51	29.81 (9)	C7—C8—C9—C10	−0.9 (3)
C49—Ru2—Ru3—C51	−143.23 (9)	C8—C9—C10—C11	−0.9 (3)
P1—Ru2—Ru3—C51	120.58 (6)	C9—C10—C11—C12	1.0 (4)
Ru1—Ru2—Ru3—C51	−70.03 (6)	C10—C11—C12—C7	0.7 (3)
C50—Ru2—Ru3—C53	148.5 (2)	C8—C7—C12—C11	−2.5 (3)
C48—Ru2—Ru3—C53	−160.52 (8)	P1—C7—C12—C11	174.19 (17)
C49—Ru2—Ru3—C53	26.44 (8)	C7—P1—C13—P2	−82.10 (12)
P1—Ru2—Ru3—C53	−69.75 (6)	C1—P1—C13—P2	169.55 (10)
Ru1—Ru2—Ru3—C53	99.64 (6)	Ru2—P1—C13—P2	45.71 (11)
C50—Ru2—Ru3—P2	−114.7 (2)	C20—P2—C13—P1	−145.16 (10)
C48—Ru2—Ru3—P2	−63.73 (6)	C14—P2—C13—P1	112.03 (11)
C49—Ru2—Ru3—P2	123.23 (6)	Ru3—P2—C13—P1	−19.42 (12)
P1—Ru2—Ru3—P2	27.042 (17)	C20—P2—C14—C15	138.44 (18)
Ru1—Ru2—Ru3—P2	−163.569 (13)	C13—P2—C14—C15	−116.75 (18)
C50—Ru2—Ru3—Ru1	48.9 (2)	Ru3—P2—C14—C15	12.1 (2)
C48—Ru2—Ru3—Ru1	99.84 (6)	C20—P2—C14—C19	−43.90 (18)
C49—Ru2—Ru3—Ru1	−73.20 (6)	C13—P2—C14—C19	60.91 (18)
P1—Ru2—Ru3—Ru1	−169.389 (13)	Ru3—P2—C14—C19	−170.27 (14)
C46—Ru1—Ru3—C52	−149.96 (12)	C19—C14—C15—C16	−1.7 (3)
C47—Ru1—Ru3—C52	−67.31 (10)	P2—C14—C15—C16	176.00 (17)
C45—Ru1—Ru3—C52	111.01 (9)	C14—C15—C16—C17	0.9 (4)
P3—Ru1—Ru3—C52	15.90 (7)	C15—C16—C17—C18	0.8 (4)
Ru2—Ru1—Ru3—C52	−171.78 (7)	C16—C17—C18—C19	−1.8 (4)
C46—Ru1—Ru3—C51	125.40 (12)	C17—C18—C19—C14	1.0 (3)
C47—Ru1—Ru3—C51	−151.95 (10)	C15—C14—C19—C18	0.7 (3)
C45—Ru1—Ru3—C51	26.37 (9)	P2—C14—C19—C18	−177.03 (17)
P3—Ru1—Ru3—C51	−68.75 (7)	C14—P2—C20—C21	−80.89 (17)
Ru2—Ru1—Ru3—C51	103.57 (7)	C13—P2—C20—C21	173.86 (15)
C46—Ru1—Ru3—C53	−55.23 (12)	Ru3—P2—C20—C21	48.24 (17)
C47—Ru1—Ru3—C53	27.42 (9)	C14—P2—C20—C25	96.33 (18)
C45—Ru1—Ru3—C53	−154.26 (9)	C13—P2—C20—C25	−8.92 (19)
P3—Ru1—Ru3—C53	110.62 (6)	Ru3—P2—C20—C25	−134.54 (16)
Ru2—Ru1—Ru3—C53	−77.06 (6)	C25—C20—C21—C22	1.0 (3)
C46—Ru1—Ru3—P2	52.87 (11)	P2—C20—C21—C22	178.35 (16)
C47—Ru1—Ru3—P2	135.52 (7)	C20—C21—C22—C23	−0.6 (3)
C45—Ru1—Ru3—P2	−46.16 (7)	C21—C22—C23—C24	0.1 (3)
P3—Ru1—Ru3—P2	−141.27 (3)	C22—C23—C24—C25	0.0 (3)
Ru2—Ru1—Ru3—P2	31.04 (3)	C23—C24—C25—C20	0.3 (3)
C46—Ru1—Ru3—Ru2	21.83 (10)	C21—C20—C25—C24	−0.8 (3)
C47—Ru1—Ru3—Ru2	104.47 (7)	P2—C20—C25—C24	−178.00 (16)
C45—Ru1—Ru3—Ru2	−77.20 (6)	C38—P3—C26—C31	−168.44 (16)
P3—Ru1—Ru3—Ru2	−172.320 (16)	C32—P3—C26—C31	84.83 (17)
C50—Ru2—P1—C7	−112.21 (10)	Ru1—P3—C26—C31	−43.23 (18)
C48—Ru2—P1—C7	157.30 (10)	C38—P3—C26—C27	12.31 (19)
C49—Ru2—P1—C7	−19.60 (10)	C32—P3—C26—C27	−94.41 (18)
Ru1—Ru2—P1—C7	58.87 (8)	Ru1—P3—C26—C27	137.52 (15)
Ru3—Ru2—P1—C7	77.45 (8)	C31—C26—C27—C28	0.6 (3)
C50—Ru2—P1—C1	14.61 (10)	P3—C26—C27—C28	179.80 (17)
C48—Ru2—P1—C1	−75.89 (9)	C26—C27—C28—C29	−0.3 (3)
C49—Ru2—P1—C1	107.22 (9)	C27—C28—C29—C30	0.2 (3)
Ru1—Ru2—P1—C1	−174.31 (7)	C28—C29—C30—C31	−0.4 (3)
Ru3—Ru2—P1—C1	−155.74 (7)	C29—C30—C31—C26	0.7 (3)
C50—Ru2—P1—C13	127.26 (9)	C27—C26—C31—C30	−0.8 (3)
C48—Ru2—P1—C13	36.77 (9)	P3—C26—C31—C30	179.95 (16)
C49—Ru2—P1—C13	−140.13 (9)	C38—P3—C32—C33	99.6 (2)
Ru1—Ru2—P1—C13	−61.65 (7)	C26—P3—C32—C33	−153.73 (19)
Ru3—Ru2—P1—C13	−43.08 (6)	Ru1—P3—C32—C33	−22.2 (2)
C52—Ru3—P2—C20	−76.67 (10)	C38—P3—C32—C37	−78.7 (2)
C51—Ru3—P2—C20	15.39 (10)	C26—P3—C32—C37	27.9 (2)
C53—Ru3—P2—C20	−170.26 (10)	Ru1—P3—C32—C37	159.44 (16)
Ru2—Ru3—P2—C20	108.95 (7)	C37—C32—C33—C34	0.9 (4)
Ru1—Ru3—P2—C20	82.64 (8)	P3—C32—C33—C34	−177.5 (2)
C52—Ru3—P2—C14	41.86 (10)	C32—C33—C34—C35	−1.4 (4)
C51—Ru3—P2—C14	133.92 (10)	C33—C34—C35—C36	1.1 (4)
C53—Ru3—P2—C14	−51.73 (9)	C34—C35—C36—C37	−0.3 (4)
Ru2—Ru3—P2—C14	−132.51 (7)	C33—C32—C37—C36	−0.1 (4)
Ru1—Ru3—P2—C14	−158.83 (7)	P3—C32—C37—C36	178.3 (2)
C52—Ru3—P2—C13	164.73 (9)	C35—C36—C37—C32	−0.2 (4)
C51—Ru3—P2—C13	−103.21 (9)	C26—P3—C38—C39	−127.87 (18)
C53—Ru3—P2—C13	71.14 (9)	C32—P3—C38—C39	−21.7 (2)
Ru2—Ru3—P2—C13	−9.64 (7)	Ru1—P3—C38—C39	101.86 (17)
Ru1—Ru3—P2—C13	−35.96 (8)	C26—P3—C38—C43	57.53 (19)
C46—Ru1—P3—C38	−68.34 (10)	C32—P3—C38—C43	163.74 (17)
C47—Ru1—P3—C38	−163.62 (10)	Ru1—P3—C38—C43	−72.75 (18)
C45—Ru1—P3—C38	22.60 (9)	C43—C38—C39—C40	1.8 (3)
Ru3—Ru1—P3—C38	120.10 (7)	P3—C38—C39—C40	−172.90 (17)
C46—Ru1—P3—C26	170.85 (10)	C38—C39—C40—C41	−1.6 (3)
C47—Ru1—P3—C26	75.57 (10)	C39—C40—C41—C42	−0.1 (3)
C45—Ru1—P3—C26	−98.21 (10)	C39—C40—C41—S1	176.83 (17)
Ru3—Ru1—P3—C26	−0.72 (8)	C44—S1—C41—C40	169.09 (19)
C46—Ru1—P3—C32	48.50 (9)	C44—S1—C41—C42	−14.1 (2)
C47—Ru1—P3—C32	−46.77 (10)	C40—C41—C42—C43	1.6 (3)
C45—Ru1—P3—C32	139.44 (9)	S1—C41—C42—C43	−175.09 (17)
Ru3—Ru1—P3—C32	−123.06 (7)	C41—C42—C43—C38	−1.4 (3)
C7—P1—C1—C6	−5.4 (2)	C39—C38—C43—C42	−0.3 (3)
C13—P1—C1—C6	103.55 (19)	P3—C38—C43—C42	174.42 (17)
Ru2—P1—C1—C6	−138.36 (16)		

Symmetry codes: (i) −*x*, −*y*+2, −*z*.

### Hydrogen-bond geometry (Å, °)

**Table:**

Cg1, Cg2 and Cg3 are the centroids of the C26–C31, C14–C19 and C7–C12 benzene rings, respectively.

**Table:**

*D*—H···*A*	*D*—H	H···*A*	*D*···*A*	*D*—H···*A*
C9—H9A···Cg1^ii^	0.93	2.83	3.550 (2)	135
C12—H12A···Cg2	0.93	2.98	3.743 (2)	140
C16—H16A···Cg1^iii^	0.93	2.90	3.696 (2)	145
C22—H22A···Cg3^iv^	0.93	2.99	3.708 (3)	136
C34—H34A···Cg2^v^	0.93	2.98	3.850 (3)	156

Symmetry codes: (ii) *x*−1, *y*, *z*;  (iii) −*x*+2, −*y*+2, −*z*+1; (iv) *x*+1, *y*, *z*; (v) *x*, *y*+1, *z*.
